# Transcutaneous auricular vagus nerve stimulation paired with task-specific training for improving walking and balance in chronic stroke: a double-blind, randomised controlled feasibility trial

**DOI:** 10.1186/s12984-026-01952-5

**Published:** 2026-06-25

**Authors:** Ashraf N. H. Gerges, Jeric Uy, Lewis Ingram, Susan Hillier, Joanne Bouckley, Ellana Welsby, Ines Serrada, Taya Hamilton, Saran Chamberlain, Brenton Hordacre

**Affiliations:** 1 Innovation, Implementation and Clinical Translation (IIMPACT) in Health, School of Allied Health and Human Performance, College of Health, Adelaide University, Adelaide, SA Australia; 2 Alliance for Research in Exercise, Nutrition and Activity (ARENA), School of Allied Health and Human Performance, College of Health, Adelaide University, Adelaide, SA Australia; 3 Perron Institute for Neurological and Translational Science, University of Western Australia Medical School, Perth, WA Australia; 4 School of Medical and Health Sciences, Edith Cowan University, Perth, WA Australia

## Abstract

**Background:**

Transcutaneous auricular vagus nerve stimulation (taVNS) paired with task-specific training has shown promise for improving upper-limb motor recovery after stroke but its effects on gait and balance remain unclear. This study evaluated the feasibility and safety of taVNS paired with task-specific training and explored preliminary effects on walking and balance outcomes in adults with chronic stroke.

**Methods:**

A double-blinded, sham-controlled trial was conducted to evaluate active versus sham taVNS paired with task-specific training (15 one-hour sessions over six weeks). Feasibility metrics included recruitment, participant experience, adverse events (AEs), and blinding effectiveness. Clinical outcomes (walking speed, endurance, gait parameters, and balance) were assessed at baseline, post-intervention, and ≥ 30 days post-intervention, supplemented by qualitative interviews.

**Results:**

Twenty-five adults with chronic stroke completed this study (18 males, mean age 64.99 ± 13.45 years; 41.80 ± 38.00 months post-stroke, 14 received active taVNS). Active taVNS was considered feasible and safe, with a 93% consent rate; 96% of participants found the intervention useful/helpful, and 100% found the stimulation acceptable. There were no serious AEs, with 16% of participants reporting mild side effects. Both groups improved across clinical outcomes. No statistically significant between-group differences were observed. Between-group effect sizes were small for gait, balance, disability, and self-efficacy, and small-to-moderate for walking endurance; however, confidence intervals crossed zero for all outcomes.

**Conclusion:**

Active taVNS paired with task-specific training was feasible and well tolerated in chronic stroke. No definitive between-group effects were detected. Observed effect sizes should inform the design and sample size calculations of future adequately powered trials using optimised taVNS protocols.

**Supplementary Information:**

The online version contains supplementary material available at 10.1186/s12984-026-01952-5.

## Introduction

Stroke is the third-leading cause of death and disability (combined) in the world [1]. The 2019 Global Burden of Disease report recorded approximately 12.2 million incidents of stroke, with 143 million disability-adjusted life-years lost [2]. Task-specific training improves motor function post-stroke, likely by driving activity-dependent neuroplasticity [3]. Task-specific training leads to greater recovery in the early stage (≤ 3 months after stroke) [4], corresponding to a period of heightened neuroplasticity [5], compared to the chronic stage (≥ 6 months after stroke) [4]. This highlights the need for adjunct therapies to augment rehabilitation in chronic stroke through enhancing neuroplasticity.

Vagus nerve stimulation (VNS) is an emerging neuromodulation approach that may augment rehabilitation outcomes after stroke [6]. Systematic reviews reported that VNS paired with upper limb motor training in people with stroke led to greater improvements in motor function compared to motor training alone [6, 7]. Clinically, VNS can be delivered invasively via a surgically implanted stimulator targeting the cervical vagus nerve (invasive VNS; iVNS), or non-invasively using transcutaneous auricular VNS (taVNS), applied to the external ear. Although both modalities aim to engage vagal pathways [8, 9], their effects are not necessarily directly comparable because stimulation sites and parameter settings differ substantially across techniques [10]. While iVNS delivers stimulation directly to the cervical vagus nerve and is relatively safe, taVNS is generally more accessible, costs less, and helps to avoid potential risks and complications associated with device implantation surgery [11–13].

Vagus nerve stimulation is thought to enhance stroke rehabilitation by engaging ascending vagal afferent pathways that modulate brain networks supporting neuroplasticity and motor learning [10, 14]. Unilateral VNS activates the nucleus tractus solitarius, a key brainstem relay with projections to bilateral neuromodulatory centres and widespread cortical and subcortical circuits [8, 9]. Through these projections, VNS can influence the release of neurotransmitters linked to synaptic plasticity, including norepinephrine, acetylcholine, and serotonin, and can also shift inhibitory signalling via GABA_A_-mediated circuits [14–19]. In humans, elevated serotonergic signalling has been linked to enhanced neuroplasticity, including changes in synaptic plasticity and adult neurogenesis [20]. Both noradrenergic and cholinergic systems have been shown to facilitate neuroplasticity through long-term potentiation [21, 22]. Human studies using taVNS report changes consistent with increased engagement of GABA_A_-related inhibitory networks [23–25]. By modulating these neurotransmitters, VNS may facilitate activity-dependent plasticity when paired with training [14, 16–18]. Preclinical stroke models provide complementary evidence as iVNS paired with motor training has led to a six-fold increase in synaptic connectivity in the lesioned hemisphere and behavioural gains that persist for two months beyond the stimulation period [26]. Together, these findings provide a mechanistic rationale for pairing VNS with task-specific training to support motor recovery. Furthermore, evidence from functional magnetic resonance imaging studies shows that taVNS activates brainstem vagal afferent projection and widespread cortical and subcortical networks [8, 9]. This widespread neuromodulatory effect of taVNS makes it well-suited to target motor behaviours that rely on bilateral and deeper cortical and subcortical networks, which are difficult to influence using focal, surface-targeted techniques such as repetitive transcranial magnetic stimulation or transcranial direct current stimulation.

Recovery of walking after stroke might be a suitable therapeutic target for taVNS, given that it is a motor behaviour that requires coordinated interaction across cortical, subcortical, brainstem, and spinal circuits [27] that could be targeted by taVNS. Preliminary clinical observations are supportive, for example, a case report described improvements in self-paced and fast gait speed and six-minute walk distance when iVNS was paired with daily walking or cycling practice over several months, with the largest gains evident at later follow-up [28]. Emerging evidence suggests that taVNS can enhance upper-limb outcomes when delivered with task-specific training [29]. However, because locomotion is bilaterally controlled and differs from many upper-limb tasks, responsiveness may not generalise directly, and dedicated mobility-focused trials are required. Because balance impairment is common after stroke and is a key contributor to falls and mobility limitation [30], we evaluated balance alongside gait to capture clinically meaningful changes in safe ambulation and fall risk. Accordingly, we aimed to assess the feasibility, safety, and preliminary efficacy of taVNS paired with task-specific gait and balance training in people with chronic stroke, in the first randomised controlled trial (RCT) of this approach [28]. We hypothesised that this intervention would be feasible and safe.

## Methods

### Participants

Adults (18 years or older) were invited to participate if they had a first-ever haemorrhagic or ischaemic stroke ≥ six months prior, could walk 10 m with or without a gait aid or assistance, had no history of other neurological conditions, had impaired gait and balance, were naive to taVNS and were safe for brain stimulation [31]. Participants were excluded if they had a history of vagus nerve surgery (i.e. vagotomy), moderate to severe cognitive or language impairment (MoCA score of less than 18), severe pain in any of the affected limb joints, advanced cardiac, pulmonary, kidney or liver condition, active implants, cerebral shunts, open skin at the point of stimulation or were pregnant. Participants were recruited from the University of South Australia (UniSA) Health and Medical Clinic, through social media advertising, and advertisement on the Stroke Foundation website. All assessments and treatment sessions were conducted within the UniSA Health and Medical Clinic, Adelaide, Australia. The study was approved by the UniSA Human Research Ethics Committee (HREC: 205153). The study protocol was pre-registered on the Australian New Zealand Clinical Trial Registry on the 13th of April 2023, trial ID: ACTRN12623000376640. The first participant was enrolled into the study on the 9th of May 2023. As a feasibility study, a power calculation was not required; we followed guidelines that recommended a number between 12 and 30 participants for feasibility studies [32].

### Study design

This was a double-blinded, sham-controlled trial, with concealed random allocation to active or sham taVNS. Trial conduct followed the Consolidated Standards of Reporting Trials (CONSORT) framework extension for randomised pilot and feasibility trials [33]. All participants received 15x one-hour physiotherapy sessions conducted over six weeks (two to three sessions per week). Active or sham taVNS was paired with task-specific walking and balance training. The task-specific training was tailored to participants’ goals and impairment levels. taVNS was delivered concurrently with physiotherapy, with stimulation commencing at the start of each treatment session and ceasing at the end. Participants were randomly assigned (1:1 by an independent researcher, https://www.randomizer.org/) to the active or sham taVNS group and were enrolled in consecutive order. Randomisation codes were concealed until the day of the first intervention. Participants and the outcome assessors were blind to allocation. The physiotherapist who provided the intervention was unblinded due to the practical requirements of delivering stimulation. However, several strategies were implemented to minimise potential bias. A structured script was used during stimulation setup to ensure consistent communication across participants, a standardised treatment framework was followed, and session duration was fixed (60 min),

### Intervention

#### Transcutaneous auricular vagus nerve stimulation

**Fig. 1:**
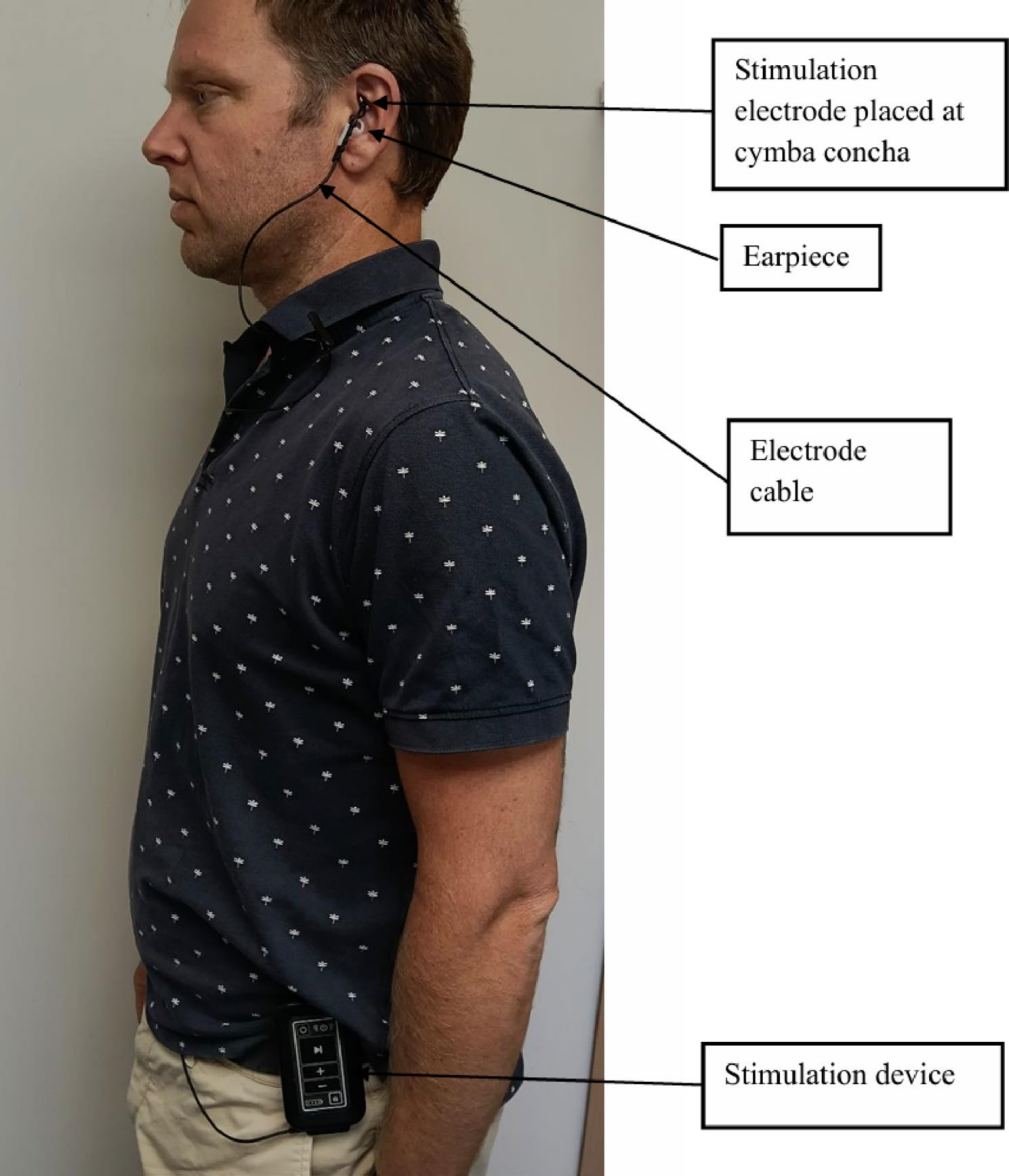
Transcutaneous auricular vagus nerve stimulation (taVNS) setup. This figure illustrates the taVNS system configuration. The stimulation electrode is positioned at the cymba concha of the left ear and connected via a lead cable to a portable stimulation control unit, which is clipped at the waist

taVNS was performed using a tVNS-R device (tVNS Technologies, Bayern, Germany) to deliver a bi-phasic waveform. An earpiece carrying two titanium/titanium-iridium hemispheric electrodes with conductive paste was placed in the participant’s left ear in contact with the cymba concha (Fig. 1). Left-ear stimulation was used because VNS research commonly avoids right-ear stimulation as it carries greater risks to cardiac side effects such as bradycardia and syncope [34]. For example, a large systematic review of taVNS trials reported that 80.01% of the studies included in the review delivered stimulation to the left ear [35]. Detection threshold was set at an intensity where participants perceived stimuli (e.g. tingling, warmth, pricking/stinging, or vibration) after incrementally increasing by 0.1 mA. Maximal tolerable intensity was determined as the intensity at which participants reported discomfort or pain at the electrode site, then lowered by 0.1 mA increments until the sensation became tolerable. The maximal tolerable intensity level was used in intervention sessions of the active taVNS group [36, 37]. The mean taVNS stimulation intensity was 2.64 mA (range 1.3–4.9 mA). The pulse width was 0.25 ms, pulse frequency was 25 Hz, and the pulse-pause ratio was 30 s on and 30 s off [11]. For the sham group, electrodes were also attached to the cymba concha of the left ear, but the stimulation device was switched off, and no electric current was delivered [23]. During each session, participants were asked to report the sensation after the intervention started and to inform the researcher if this changed throughout the intervention. During active taVNS, if a participant reported feeling nothing, the researcher verified the device setup to ensure connectivity and electrode contact with the skin until the participant perceived the stimulus again. For sham taVNS, when a participant reported no sensation, the researcher reassured the participant of proper device connectivity and reminded them that sensation could vary depending on stimulation parameters. taVNS device activity was monitored during all sessions to ensure the device was functioning appropriately.

#### Physiotherapy

The physiotherapy (task-specific training) component of the intervention was based on the Stroke Foundation Clinical Guidelines (Australia) for Stroke Management and delivered by a registered physiotherapist. A framework based on motor learning principles was followed, and intervention was tailored to individuals’ impairments, rehabilitation goals, and level of mobility. Sessions included the following components: (1) goal-oriented task-specific training (for example, practice of walking components followed by practice of walking overground or on a treadmill, and balance training in a standing position), (2) aerobic exercise (for example, bicycle ergometer, walking on a treadmill or overground), (3) core and lower limbs strengthening exercises, and (4) lower limb passive/active stretching.

#### Demographics and clinical characteristics

Age, sex, date of stroke, type of stroke (ischemic/haemorrhagic), handedness (self-reported) and active prescription of neuroactive medication were collected during screening. Current level of mobility was documented using Functional Ambulation Categories (FAC) [38]. The FAC is a six-point functional walking test; zero means nonfunctional ambulator, and five means independent ambulator.

### Outcome measures

#### Feasibility and safety

A-priori feasibility criteria were evaluated by measuring consent rates, adherence, acceptability, and satisfaction with the intervention (determined using a five-point Likert Scale [39]) (refer to supplementary file). AEs and blinding were evaluated using questionnaires [11] (refer to supplementary file), with falls or near misses during the six weeks of intervention documented in a falls diary (refer to supplementary file). Adherence was monitored by auditing participants’ attendance checklists. If all feasibility criteria (described in the supplementary file) met the requirement to “proceed” or “proceed with protocol amendment “, then the study would be deemed feasible.

#### Preliminary efficacy

Six clinical measures were recorded at baseline, post-intervention (within 5 days) and follow-up (≥ 30 days post-intervention). A 10-m walk test (10MWT) was used to assess (fast) walking speed [40]. Participants were asked to walk a 10 m distance as fast as possible. The time taken to walk the middle 6 m was recorded and repeated three times, and the average speed (m/s) was used in the analysis. The 10MWT is a valid measure to evaluate either self-paced or fast-walking speed [41] and has excellent inter-rater reliability (interclass correlation coefficient (ICC) = 0.97) in people with chronic stroke [42]. The two-Minute Walk Test (2MWT) was used to assess walking endurance. It measured the maximum distance walked by an individual in two minutes. This measure is shown to be valid to estimate cardiorespiratory fitness in people with chronic stroke [43]. The 2MWT has good intra-*rater* and inter-rater reliability (ICC = 0.85 for both) in stroke [44]. GAITRite^®^ (overall mat length 7 m) was used to assess spatiotemporal parameters of gait, including self-paced gait speed (m/s), step length (cm), cadence (steps/minute), % of swing phase and stance phase on gait cycle, step time (s), single and double support time (s). The Brief Balance Evaluation System Test (BESTest) was used to evaluate balance. This measure was selected because it provides an efficient, systems-based assessment of balance that samples six postural control domains: biomechanical constraints, stability limits/verticality, anticipatory adjustments, reactive responses, sensory orientation, and gait stability, which aligns with the multisystem balance deficits commonly seen after stroke [45]. Moreover, it has no substantial floor or ceiling effect in people with chronic stroke [45]. It has good intra-rater (ICC = 0.974) and inter-rater (ICC = 0.980) reliability and internal consistency (Cronbach’s alpha = 0.818) in people with chronic stroke [45]. Modified Rankin Score (mRS) is a clinician-reported measure of global disability; it is a valid and reliable measure with moderate inter-rater reliability (κ = 0.56) and strong test-retest reliability in people with chronic stroke (κ = 0.8) [46]. The Stroke Self-Efficacy Questionnaire (SSEQ) is a 13-item self-report measure that assesses a person’s confidence (self-efficacy) in performing functional activities and aspects of self-management after stroke; items are rated on a confidence scale, with higher scores indicating greater self-efficacy [47]. It is valid and reliable for assessing individuals with chronic stroke [48]. MDC for 2MWT was 23% (percentage of baseline measure) [49]. The MDC for fast and self-paced gait speed varied depending on participants’ baseline gait (slow walkers < 0.4 m/s, moderate speed walkers 0.4 to 0.8 m/s, and fast walkers > 0.8 m/s). For fast gait speed, MDCs were 0.11, 0.17, 0.19, and for self-paced gait speed, MDCs were 0.10, 0.15, and 0.18, respectively [50]. The MDC for Brief BESTest was two points [45]. The MCID of SSEQ was 3.7 points [48].

Semi-structured individual interviews were held with the participants (*n* = 25) after the completion of the trial. The interview guide included nine questions (refer to supplementary file). These qualitative data were gathered to help inform future research about treatment design, participants ‘acceptance, and compliance with taVNS. The interviews were conducted in person with the trial participants and with help from interpreters for those who had limited English (n = 2) by an interviewer who was blind to the intervention.

### Analysis

Participants' demographics and baseline clinical measures were compared between groups using independent-samples t-tests for continuous variables (age, time since stroke, FAC, Montreal Cognitive Assessment (MoCA) [51], mRS, SSEQ, Brief BESTest, 10MWT, 2MWT, and self-paced gait speed). Fisher's exact test was used to compare categorical variables, including sex, stroke type, handedness, and affected side.. The feasibility criteria were reported against pre-specified thresholds (refer to supplementary file). Safety and blinding outcomes were summarised descriptively. Blinding was considered successful when approximately equal numbers of participants believed they received the active intervention in each condition (i.e., balanced beliefs about allocation) [52]. Exploratory analyses of blinding efficacy were computed using Bang’s blinding index (BI) [52]. Bang’s BI values range from − 1.00 to + 1.00 [52]. If all blinding guesses were correct, BI would equal 1.00 (complete unblinding). If all guesses were incorrect, BI equals − 1.00 (opposite guessing). A BI close to zero indicates random guessing. Moreover, unblinding may be claimed if the lower bound of the 95%CI is greater than zero [52].

For treatment efficacy, several linear mixed models analyses were performed for each measure. The dependent variables were mRS, SSEQ, 2MWT distance (m), fast gait speed (m/s), Brief BESTest (score), and gait spatiotemporal parameters measured using Gaitrite. Each model compared two groups (taVNS and sham) and three-time points (baseline, post-intervention, and follow-up). All analyses were performed in IBM SPSS Statistics for Windows, Version 28.0 [53], with the level of significance set at *p* < 0.05. Improvements in clinical measures against MCID/MDC were also compared between groups. Ratios of participants who reached or exceeded MCID/MDC were compared between groups using Fisher’s Exact test. Lastly, the effects of sex or laterality on clinical outcomes were explored using an independent t-test in the active taVNS group for change from baseline to post-intervention. These effects were explored because there is evidence indicating the sex-related difference in response to taVNS effect on excitability of the motor cortex in healthy adults, with female participants showing a greater increase in GABA_A_ergic inhibition than males in response to 60 min of taVNS stimulation delivered to cymba concha of left ear [23]. Given the exploratory objectives of this feasibility trial, no adjustment for multiple comparisons was applied.

Consistent with recommendations for clinical and rehabilitation research, effect sizes were calculated to support interpretation of clinical relevance and to inform sample size estimation for future trials [54]. Intervention effects were quantified using Cohen’s d, calculated as the standardised mean difference in change scores (post–pre) between the active taVNS and sham groups [55]. Ninety-five per cent confidence intervals were calculated to indicate the precision of effect estimates. Effect sizes were interpreted using conventional thresholds widely applied in stroke rehabilitation research (≈ 0.2 small, ≈ 0.5 moderate, ≥ 0.8 large) [55].

Thematic analysis was conducted for the semi-structured interviews. All interviews were transcribed and entered into an Excel spreadsheet. Open coding of the transcribed experiences was undertaken by a researcher not involved in the trial, and the codes were then incorporated inductively into themes. The process was overseen by a second researcher with discussion at various points in the analysis. Researcher bias was managed by recording the interviews and the thematic analysis being conducted by a person not involved in the intervention.

## Results

### Participants demographics and clinical characteristics

**Fig. 2:**
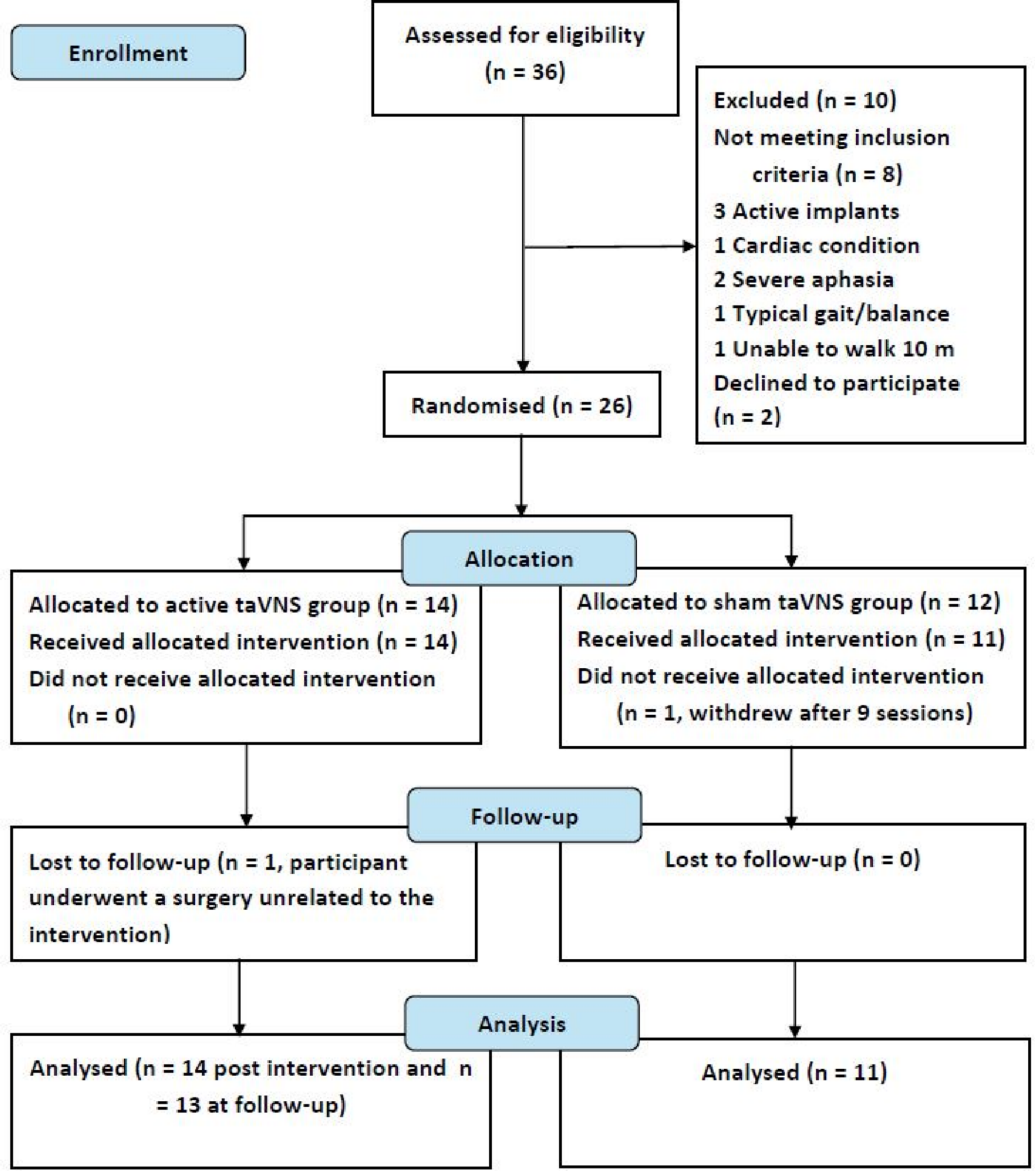
CONSORT flow diagram

Twenty-five participants (18 male, 22 right-handed, age 64.99 ± 13.45 years, 15 right hemiparesis) completed interventions, and 24 completed follow-up from May 2023 to June 2024. Fourteen participants were allocated to active taVNS and 11 to sham. A CONSORT flow diagram is provided in Fig. 2. There was no significant difference between groups for demographics or baseline clinical measures (Table 1).

**Table 1:** Participant characteristics and baseline clinical measures

Demographics and baseline clinical measures	Active taVNS	Sham taVNS	*P* values
Age (years) mean (SD)	64.00 (12.67)	66.18 (14.92)	0.67^a^
Sex (N, male/female)	11/3	7/4	0.41^b^
Stroke-type (N, Ischemic/haemorrhagic)	10/4	9/2	0.84^b^
Handedness (N, right/left)	12/2	10/1	0.69^b^
Affected side (N, right/left)	6/8	4/7	0.74^b^
FAC mean (SD)	4.00 (1.07)	3.73 (0.90)	0.51^b^
Time since stroke (months) mean (SD)	41.35 (27.28)	43.16 (50.14)	0.91^a^
MoCA mean (SD)	25.43 (3.32)	25.91 (3.21)	0.72^a^
mRS mean (SD)	2.73 (0.47)	2.93 (0.62)	0.42^a^
SSEQ mean (SD)	96.64 (22.76)	81.00 (31.80)	0.22^a^
Brief BESTest mean (SD)	10.91 (4.53)	9.14 (7.19)	0.62^a^
10MWT (Fast paced m/s) mean (SD)	0.82 (0.36)	0.81(0.52)	0.97^a^
2MWT (m) mean (SD)	72.10 (33.74)	72.60 (42.44)	0.85^a^
Gait speed (self-paced m/s) mean (SD)	0.61 (0.23)	0.66 (0.33)	0.64^a^

taVNS: transcutaneous auricular vagus nerve stimulation, N: number of participants, FAC: Functional ambulation scale, MoCA: Montreal cognitive assessment test, mRS: modified Rankin scale, SSEQ: Stroke self-efficacy questionnaire, 10MWT: 10-m walk test, BESTest: Balance evaluation system test, 2MWT: Two-Minute Walk test, SD: standard deviation

^a^Independent-samples t-test

^b^Fisher’s exact test

### Feasibility

Baseline assessments were conducted at a mean of 2.1 ± 1.6 days before the first intervention session (range 0–5 days). Post-treatment assessments were conducted at a mean of 2.5 ± 2.1 days after the final intervention session (range 0–7 days). Follow-up assessments occurred at a mean of 36.7 ± 4.3 days after treatment completion (range 30–46 days).

The consent rate was 93%, and 96% of enrolled participants adhered to the intervention protocol (completed all intervention sessions). One participant withdrew from the sham group after completing nine intervention sessions due to a change in their circumstances and inability to attend the remaining sessions. Participants’ experiences were positive, with 96% considering it useful/helpful and 100% finding the intervention acceptable. All participants tolerated the intervention well. Sixteen per cent of participants reported mild AEs with no serious AEs. Four participants (in the taVNS group) reported AEs, including pain, irritation, itchiness at the stimulation site, headaches and fatigue. All AEs were mild and transient (refer to supplementary file for details). During the trial, the participants reported 19 near-misses (trip, slip or loss of balance without falling) and nine falls. All reported falls and near misses occurred outside the intervention sessions. The sham taVNS group reported more near-misses (*n* = 14) and falls (*n* = 5) compared to the active taVNS group (four falls and five near-misses). There were no significant differences between active and sham groups for number of falls (*p* = 0.88). Of note, 11 of the 14 near-misses reported in the sham group were reported by one participant, and most of them were from their foot catching while walking indoors and not wearing their foot-up orthosis.

Blinding was partially successful but less effective in the active taVNS group, likely due to sensory cues associated with stimulation. In the active taVNS group, 57% of participants correctly guessed their allocation, which is only slightly higher than the 50% expected by chance. In contrast, 27% of participants in the sham group guessed correctly. The Bang Blinding Index was 0.50 (95% CI: 0.17–0.83) for the active group and 0.09 (95% CI: −0.30–0.49) for the sham group (see supplementary file for details).

### Preliminary efficacy

#### Clinical outcome measures

**Fig. 3:**
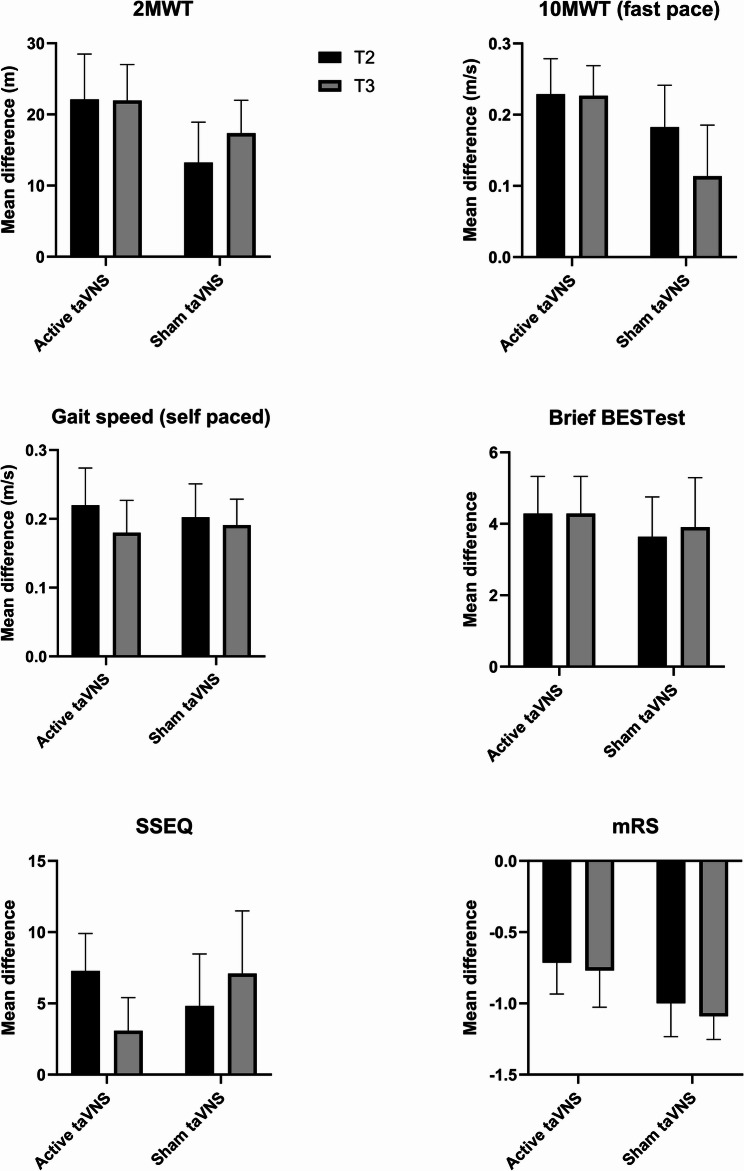
The mean difference between clinical measures at baseline and post-intervention/follow-up. T2: within 5 days post-intervention; T3: ≥ 30 days post-intervention

For 10MWT (fast paced) (m/s), 2MWT, Brief-BESTest, SSEQ and spatiotemporal parameters measures, including self-paced gait speed (m/s) and cadence, there was an effect of time but no effect of group or group*time interaction. There was no effect of time, group or time*group interaction on the remaining spatiotemporal gait measures of the affected or unaffected lower limb. For mRS there was an effect of time and of group but no effect of group*time interaction (Table 2; Fig. 3).

**Table 2:** Effect of taVNS on clinical outcome measures

Outcome measure	Type III Tests of Fixed Effects		
	Source	F	Sig.
mRS	Time	12.81	**< 0.001**
	Condition	4.15	**0.05**
	Time * Condition	0.36	0.67
SSEQ	Time	4.01	**0.03**
	Condition	1.58	0.22
	Time * Condition	1.86	0.18
2MWT (m)	Time	17.30	**< 0.001**
	Condition	0.16	0.70
	Time * Condition	0.60	0.56
Brief BESTest	Time	18.82	**< 0.001**
	Condition	0.25	0.62
	Time * Condition	0.20	0.82
10MWT (fast-paced) (m/s)	Time	18.06	**< 0.001**
	Condition	0.11	0.74
	Time * Condition	1.25	0.31
Self-paced gait speed (m/s)	Time	20.76	**< 0.001**
	Condition	0.08	0.79
	Time * Condition	0.43	0.66
Cadence	Time	25.58	**< 0.001**
	Condition	0.011	0.92
	Time * Condition	0.18	0.84
Double support time (s)	Time	0.76	0.47
	Condition	0.44	0.51
	Time * Condition	0.00	0.99
*Unaffected lower limb*			
Step time	Time	0.51	0.60
	Condition	0.35	0.55
	Time * Condition	0.04	0.96
Step length	Time	2.56	0.09
	Condition	0.96	0.33
	Time * Condition	0.02	0.98
Single support time (s)	Time	0.65	0.53
	Condition	2.07	0.16
	Time * Condition	0.02	0.98
Swing % of the gait cycle	Time	0.71	0.50
	Condition	0.44	0.51
	Time * Condition	0.01	0.99
Stance % of gait cycle	Time	1.33	0.28
	Condition	0.09	0.77
	Time * Condition	0.02	0.98
*Affected lower limb*			
Step time	Time	0.67	0.52
	Condition	0.01	0.92
	Time * Condition	0.01	0.99
Step length	Time	1.70	0.20
	Condition	0.34	0.56
	Time * Condition	0.04	0.97
Single support time	Time	0.10	0.91
	Condition	0.01	0.91
	Time * Condition	0.02	0.98
Swing % of gait cycle	Time	0.78	0.47
	Condition	1.44	0.24
	Time * Condition	0.03	0.97
Stance % of gait cycle	Time	0.79	0.46
	Condition	1.45	0.23
	Time * Condition	0.03	0.97

taVNS: transcutaneous auricular vagus nerve stimulation, mRS: modified Rankin scale, SSEQ: Stroke self-efficacy questionnaire, 10MWT: 10-m walk test, BESTest: Balance evaluation system test, 2MWT: Two-minute walk test, Bold values indicate statistical significance (p < 0.05).

The percentage of participants who reached or exceeded the MCID of SSEQ or MDC for 10MWT, 2MWT and self-paced gait speed was greater in the active taVNS than in the sham, but the difference did not reach significance (Table 3).

**Table 3:** The percentage of participants who reached or exceeded minimal detectable change or minimal clinical important difference of clinical measures with Exact Fisher’s Test results for a comparison between active and sham taVNS groups

Measure	Post-intervention				Follow-up			
	Active taVNS %	Sham taVNS %	Exact sig. (2-sided)	*N* active/sham	Active taVNS %	Sham taVNS %	Exact sig. (2-sided)	*N* active/sham
SSEQ	64	55	0.70	14/11	46	53	0.44	13/11
Brief BESTest	71	72	1.00	14/11	46	53	0.44	13/11
10MWT (fast-paced m/s)	78	63	0.66	14/11	71	45	0.21	13/11
2MWT (m)	57	45	0.44	14/11	69	54	0.68	13/11
Self-paced gait speed (m/s)	69	63	1.00	13/11	58	73	0.67	12/11

m: Meters, SSEQ: Stroke Self-Efficacy Scale, N: number of participants, 10MWT: 10-m walk test, 2MWT: 2-min walk test

Compared with sham stimulation, the active taVNS intervention was associated with small effect sizes for mRS (Cohen’s d = 0.36, 95% CI − 0.44 to 1.15), SSEQ (Cohen’s d = 0.23, 95% CI − 0.57 to 1.02), fast-paced walking speed (10MWT) (Cohen’s d = 0.24, 95% CI − 0.550 to 1.035), Brief BESTest (Cohen’ = 0.17, 95% CI − 0.62 to 0.96), cadence (Cohen’s d = 0.21, 95% CI − 0.58 to 1.00), and self-paced gait velocity (Cohen’s d = 0.21, 95% CI − 0.59 to 1.02). Small-to-moderate effects were observed for walking endurance measured by the 2MWT (Cohen’s d = 0.42, 95% CI − 0.38 to 1.22). There was no significant difference for any of the clinical measures between participants with left and right affected sides or male and female participants (all *p* > 0.05) (refer to supplementary file for details).

#### Participants experience

Three themes emerged: (A) a positive experience, whether we could feel it or not; (B) benefits were varied and hard to attribute; (C) being part of discovery of the traditional pathways.

##### A positive experience

Overwhelmingly, participants found being in the trial to be a “good” experience. Often, this was qualified by the observation that they could not feel the ear stimulation (all sham participants) or that if they could feel it, it was only a buzzing feeling that faded to unnoticeable as they did other activities (typical of the intervention group).

“I found it good. Like I said, you feel it at first and then as you get on with the task you doing it becomes less and less obvious to you.” P02.

No participants reported a negative experience – a few had the stimulation intensity higher than usual on one occasion, but it felt more like a surprise than a negative sensation. All participants felt happy to attend all sessions and only missed if something else interfered with their attendance. However, they were able to make up for any missed sessions during the six weeks of intervention.

##### Benefits were varied

Most reported that they believed they received benefits from the trial. Some felt the stimulation did add to their observed improvements, but most were open-minded about what aspects of the trial contributed to their benefits. Often, the benefits were expressed as increased confidence.

“I feel like my confidence has increased greatly and I find that I’m walking a lot smoother and going more of a distance and I’m not so uneven, like feeling really uneasy and feeling really scared?” P04.

“it’s given me a lot more confidence. And I already said, like I’m going out on the weekend with my girlfriend and normally I just choose not to because I don’t like being out and around in public. But I also sort of feel, you know, and it’s just because I put up walls before they’re even there. But yeah…I’m very keen to keep progressing with what I’ve learned and the tools I’ve been given. Yeah, just to see what also keeps happening into the future.” P26.

The questions focused on improvements in balance or mobility, which several participants confirmed. Because of the multifaceted nature of the intervention, the ‘causation’ of the effect was moot.

“I don’t know whether it’s [the] stimulation but just this um um trial I’ve done, it’s definitely, definitely helped. Like we’ve done a lot of balance stuff and it’s been pretty hard work. But, yeah, I’ve definitely seen a lot of improvement in the last five weeks.” P10.

##### Being part of discovery

Several participants from both groups found great value in simply being part of a discovery process, having the opportunity to engage in something new and “innovative”. This was sometimes in contrast to their experiences in a more traditional medical model.

“I like the fact of trying new things.” P02.

## Discussion

The primary aim of this study was to evaluate the feasibility and safety of taVNS paired with walking and balance training in people with chronic stroke. The secondary aim was to gain preliminary efficacy data. Our results indicate that this intervention was feasible and safe. All participants reported a positive experience, perceived benefits, and tolerated taVNS well. Preliminary efficacy data showed that both active and sham taVNS groups improved across all clinical measures, but no statistical difference existed between groups. However, there was a trend for greater improvements in active taVNS groups with small to moderate effect sizes across all clinical measures. A greater proportion of participants in the active taVNS group reached or exceeded MCID or MDC post-intervention. Sham protocols need modification to improve blinding.

Feasibility was evaluated by seven criteria measuring consent rates, adherence, satisfaction with the intervention, acceptability, safety, and blinding efficacy. Most criteria were deemed as “proceed” to trial, except for blinding which was considered as “proceed with protocol amendment”. The consent and attrition rates (93% and 96%, respectively) in this study were comparable to those reported in RCTs investigating the taVNS effect paired with task-specific training on upper limb function in chronic stroke (85% [56] and 80% [57] to 94% [56], respectively). The intervention was safe, and the percentage of participants reporting AEs (16%) was comparable to that reported in studies that investigated the taVNS effect on upper-limb function in chronic stroke, which ranged from 0% [57, 58] to 25% [59]. All AEs were mild, transient and similar to those reported in previous studies applying taVNS with stroke survivors, including light-headedness and fatigue [37]. In our study, the active taVNS group reported fewer near-misses and falls than the sham group. Although the difference was not statistically significant and should be interpreted cautiously, this should be further investigated in future studies to explore whether taVNS may improve falls risk. The high consent and tolerability of taVNS in this study, along with positive experiences, indicate that combining taVNS with walking and balance training post-stroke is feasible and safe.

Blinding participants in taVNS research appears to be challenging and is often poorly reported or evaluated [11]. In this study, there was an imbalance in beliefs about the intervention between the active and sham taVNS groups, with participants in the active group being twice as accurate in identifying their intervention. This discrepancy is likely due to the distinct sensory experiences of active stimulation (e.g., tingling). Although this sham protocol is commonly used in taVNS trials [60–65] and aligns with previous findings [23], further optimisation is required to improve blinding, particularly given the challenge of masking the sensory effects of active interventions. Alternatives include delivering stimulation to non-vagal areas, such as the earlobe, which provides similar sensations without introducing confounding physiological effects. A recent study confirmed that earlobe stimulation and no stimulation at the cymba concha produced comparable effects on cortical excitability, which differed significantly from active stimulation at the cymba concha [24]. While that study utilised continuous stimulation [24], we propose using transient stimulation (e.g., 10–30 s every 5–10 min) as a potential approach to enhance blinding and minimise confounding effects.

Compared with prior upper-limb taVNS studies, the effects observed here for gait and balance were more modest. For example, one small study (*n* = 12) reported an effect size of Cohen’s d = 0.6 for the Fugl Meyer [59]. This discrepancy may be partly explained by the differences in the underlying neural control of upper-limb versus locomotor behaviours. Upper-limb taVNS trials have typically targeted highly repetitive, discrete movements (often delivered with robotics or task-oriented practice), where stimulation can be delivered in close temporal association with movement onset [57, 58]. More recent work further suggests that pairing precision matters, in a double blind pilot RCT, closed-loop “motor-activated” taVNS has shown larger gains in upper limb function than unpaired stimulation [57]. In contrast, locomotion is generated by bilaterally organised, distributed circuits spanning cortical, subcortical, brainstem and spinal networks [29]. Also, gait and balance training can be more variable and less discretised than upper-limb practice, which may reduce the neuromodulatory “signal-to-noise” achievable with fixed or session-level stimulation schedules. Accordingly, we speculate that optimising event-locked delivery during stepping or balance reactions (e.g., sensor-triggered stimulation during gait events) and increasing the cumulative dose of precisely paired stimulation may be particularly important for mobility outcomes. Based on our data, we calculated the sample size required for future trials. It appears 92 participants per group would be needed to achieve a statistical power of 0.8 and *p* < 0.05, based on 2MWT (Cohen’s d = 0.42). These mechanistic hypotheses remain to be tested directly in adequately powered trials that standardise training dose and explicitly manipulate stimulation timing.

There are limitations to this study. First, this was a feasibility study with a small sample size, which may lead to type 2 errors. Second, our blinding effectiveness data revealed an imbalance in participant beliefs about active versus sham conditions, which could have introduced expectancy effects (e.g., differential motivation, confidence, or engagement with training) and thereby influenced both self-reported outcomes and performance during training. Third, the treating physiotherapist was not blinded to allocation, which may have introduced expectancy bias despite standardisation procedures to minimise bias. Fourth, training dose was not objectively quantified (e.g., step counts, walking duration or repetitions), limiting interpretation of dose–response effects. Future trials should incorporate wearable sensors to capture objective step counts and should also include measures of training intensity. Fifth, follow-up period (one month post-intervention) was short which may limit ability to determine whether improvements in mobility outcomes were sustained over time; future trials should incorporate longer follow-up durations (e.g., 3–6 months) to evaluate durability of intervention effects. Lastly, this feasibility study was not designed or powered for definitive hypothesis testing. Multiple gait outcomes were examined to explore potential signals of change and to inform outcome selection for future adequately powered trials and this increased the risk of type I error. Accordingly, the findings should be interpreted with caution and considered hypothesis-generating rather than confirmatory.

Several recommendations for future trials are provided. First, future trials should test whether movement-triggered (closed-loop) taVNS enhances training effects, consistent with paired-stimulation principles. In a double-blind pilot RCT in chronic stroke, motor-activated taVNS triggered within 100 ms of electromyography-detected upper-limb activation produced numerically larger Fugl-Meyer upper-extremity gains than the same stimulation delivered during therapy but not movement-locked [57]. Extending this approach to gait and balance, wearable sensors could be used to detect stepping in real time to trigger stimulation and to objectively quantify training dose (e.g., step counts) [66]. Second, delivering home-based taVNS protocols could increase treatment dose and scalability; self-administered home taVNS was shown to be feasible and safe in other clinical populations [67]. Third, the effect size for the Brief-BESTest was slightly smaller than that observed for some gait outcomes, which may reflect limited responsiveness of this balance measure to the intervention dose or differential responsiveness of specific balance subsystems. Given the heterogeneous nature of balance, future studies may benefit from including complementary measures to capture changes across distinct balance domains. Finally, while the earpiece electrode design is easy to use and comfortable, it can detach unintentionally during participant movement. This issue can be addressed by taping electrodes to the participant’s ear to ensure they remain attached throughout intervention sessions. Alternatively, future studies could consider using an ear clip electrode design, especially when combining taVNS with walking and balance studies.

## Conclusion

Active taVNS paired with task-specific training was feasible and well tolerated, with participants reporting generally positive experiences. Small-to-moderate effect size trends were observed; however, confidence intervals crossed zero for all outcomes, indicating considerable uncertainty. Accordingly, these findings should inform the design of adequately powered trials using optimised taVNS protocols and should not be interpreted as evidence of efficacy.

## Supplementary Information

Supplementary Material 1

## Data Availability

The datasets generated and analysed during the current study are available from the corresponding author upon reasonable request.

## References

[1] Feigin VL, Brainin M, Norrving B, Martins S, Sacco RL, Hacke W, et al. World Stroke Organization (WSO): global stroke fact sheet 2022. Int J Stroke. 2022;17(1):18–29. 10.1177/17474930211065917. 34986727 10.1177/17474930211065917

[2] The rising global burden of stroke. EClinicalMedicine. 2023;59:102028. 10.1016/j.eclinm.2023.102028. 10.1016/j.eclinm.2023.102028 PMC10225620 37256100

[3] Goncalves S, Le Bourvellec M, Mandigout S, Duclos NC. Impact of active physiotherapy on physical activity level in stroke survivors: a systematic review and meta-analysis. Stroke. 2023;54(12):3097–106. 10.1161/STROKEAHA.123.043629. 37909205 10.1161/STROKEAHA.123.043629

[4] Dromerick AW, Geed S, Barth J, Brady K, Giannetti ML, Mitchell A, et al. Critical period after stroke study (CPASS): a phase II clinical trial testing an optimal time for motor recovery after stroke in humans. Proc Natl Acad Sci USA. 2021. 10.1073/pnas.2026676118. 34544853 10.1073/pnas.2026676118 PMC8488696

[5] Hordacre B, Austin D, Brown KE, Graetz L, Pareés I, De Trane S, et al. Evidence for a window of enhanced plasticity in the human motor cortex following ischemic stroke. Neurorehabil Neural Repair. 2021;35(4):307–20. 10.1177/1545968321992330. 33576318 10.1177/1545968321992330 PMC7610679

[6] Korupolu R, Miller A, Park A, Yozbatiran N. Neurorehabilitation with vagus nerve stimulation: a systematic review. Front Neurol. 2024;15:1390217. 10.3389/fneur.2024.1390217. 38872818 10.3389/fneur.2024.1390217 PMC11169586

[7] Zhao KH, Yang JE, Huang JP, Zhao ZQ, Qu Y. Effect of vagus nerve stimulation paired with rehabilitation for upper limb function improvement after stroke: a systematic review and meta-analysis of randomized controlled trials. Int J Rehabil Res. 2022;45(2):99–108. 10.1097/MRR.0000000000000509. 34839304 10.1097/MRR.0000000000000509 PMC9071025

[8] Badran BW, Dowdle LT, Mithoefer OJ, LaBate NT, Coatsworth J, Brown JC, et al. Neurophysiologic effects of transcutaneous auricular vagus nerve stimulation (taVNS) via electrical stimulation of the tragus: a concurrent taVNS/fMRI study and review. Brain Stimul. 2018;11(3):492–500. 10.1016/j.brs.2017.12.009. 29361441 10.1016/j.brs.2017.12.009 PMC6487660

[9] Frangos E, Ellrich J, Komisaruk BR. Non-invasive access to the vagus nerve central projections via electrical stimulation of the external ear: fMRI evidence in humans. Brain Stimul. 2015;8(3):624–36. 10.1016/j.brs.2014.11.018. 25573069 10.1016/j.brs.2014.11.018 PMC4458242

[10] Kaniusas E, Kampusch S, Tittgemeyer M, Panetsos F, Gines RF, Papa M, et al. Current directions in the auricular vagus nerve stimulation I: a physiological perspective. Front Neurosci. 2019;13:854. 31447643 10.3389/fnins.2019.00854 PMC6697069

[11] Gerges ANH, Williams E, Hillier S, Uy J, Hamilton T, Chamberlain S, et al. Clinical application of transcutaneous auricular vagus nerve stimulation: a scoping review. Disabil Rehabil. 2024. 10.1080/09638288.2024.2313123. 38362860 10.1080/09638288.2024.2313123

[12] Mao H, Chen Y, Ge Q, Ye L, Cheng H. Short- and long-term response of vagus nerve stimulation therapy in drug-resistant epilepsy: a systematic review and meta-analysis. Neuromodulation. 2022;25(3):327–42. 10.1111/ner.13509. 35396068 10.1111/ner.13509

[13] van Schooten J, Smeets J, van Kuijk SM, Klinkenberg S, Schijns O, Nelissen J, et al. <article-title update="added">Surgical complications of vagus nerve stimulation surgery: a 14-years single-center experience. Brain Spine. 2024;4:102733. 10.1016/j.bas.2023.102733. 38510607 10.1016/j.bas.2023.102733 PMC10951712

[14] Badran BW, Glusman CE, Badran AW, Austelle CW, DeVries WH, Borckhardt JJ, et al. The physiological and neurobiological effects of transcutaneous auricular vagus nerve stimulation (taVNS). Brain Stimul. 2017;10(2):378–88.

[15] Moller M, Mehnert J, Schroeder CF, May A. Noninvasive vagus nerve stimulation and the trigeminal autonomic reflex: an fMRI study. Neurology. 2020;94(10):e1085–93. 32029547 10.1212/WNL.0000000000008865

[16] Cheyuo C, Jacob A, Wu R, Zhou M, Coppa GF, Wang P. The parasympathetic nervous system in the quest for stroke therapeutics. J Cereb Blood Flow Metab. 2011;31(5):1187–95. 21364605 10.1038/jcbfm.2011.24 PMC3099641

[17] Frazer A, Shah A, Carreno F. Neurochemical and behavioral effects of vagal nerve stimulation. Int J Neuropsychopharmacol. 2014.

[18] Groves DA, Brown VJ. Vagal nerve stimulation: a review of its applications and potential mechanisms that mediate its clinical effects. Neurosci Biobehav Rev. 2005;29(3):493–500. 15820552 10.1016/j.neubiorev.2005.01.004

[19] Hulsey DR, Shedd CM, Sarker SF, Kilgard MP, Hays SA. Norepinephrine and serotonin are required for vagus nerve stimulation directed cortical plasticity. Exp Neurol. 2019;320:112975. 10.1016/j.expneurol.2019.112975. 31181199 10.1016/j.expneurol.2019.112975 PMC6708444

[20] Nichols JA, Nichols AR, Smirnakis SM, Engineer ND, Kilgard MP, Atzori M. Vagus nerve stimulation modulates cortical synchrony and excitability through the activation of muscarinic receptors. Neuroscience. 2011;189:207–14. 10.1016/j.neuroscience.2011.05.024. 21627982 10.1016/j.neuroscience.2011.05.024

[21] Kraus C, Castrén E, Kasper S, Lanzenberger R. Serotonin and neuroplasticity: links between molecular, functional and structural pathophysiology in depression. Neurosci Biobehav Rev. 2017;77:317–26. 10.1016/j.neubiorev.2017.03.007. 28342763 10.1016/j.neubiorev.2017.03.007

[22] Bröcher S, Artola A, Singer W. Agonists of cholinergic and noradrenergic receptors facilitate synergistically the induction of long-term potentiation in slices of rat visual cortex. Brain Res. 1992;573(1):27–36. 10.1016/0006-8993(92)90110-U. 1349501 10.1016/0006-8993(92)90110-u

[23] Katsuki H, Izumi Y, Zorumski CF. Noradrenergic regulation of synaptic plasticity in the hippocampal CA1 region. J Neurophysiol. 1997;77(6):3013–20. 10.1152/jn.1997.77.6.3013. 9212253 10.1152/jn.1997.77.6.3013

[24] Gerges ANH, Graetz L, Hillier S, Uy J, Hamilton T, Opie G, et al. Transcutaneous auricular vagus nerve stimulation modifies cortical excitability in middle-aged and older adults. Psychophysiology. 2024. 10.1111/psyp.14584. 38602055 10.1111/psyp.14584 PMC11780349

[25] Midden VM, Demšar J, Pirtošek Z, Kojović M. The effects of transcutaneous auricular vagal nerve stimulation on cortical GABAergic and cholinergic circuits: a transcranial magnetic stimulation study. Eur J Neurosci. 2023;57(12):2160–73. 10.1111/ejn.16004. 37125748 10.1111/ejn.16004

[26] Capone F, Assenza G, Di Pino G, Musumeci G, Ranieri F, Florio L, et al. The effect of transcutaneous vagus nerve stimulation on cortical excitability. J Neural Transm. 2015;122(5):679–85. 10.1007/s00702-014-1299-7. 25182412 10.1007/s00702-014-1299-7

[27] Meyers EC, Solorzano BR, James J, Ganzer PD, Lai ES, Rennaker RL 2nd, et al. Vagus nerve stimulation enhances stable plasticity and generalization of stroke recovery. Stroke. 2018;49(3):710–7. 10.1161/STROKEAHA.117.019202. 29371435 10.1161/STROKEAHA.117.019202 PMC6454573

[28] Nielsen JB. How we walk: central control of muscle activity during human walking. Neuroscientist. 2003;9(3):195–204. 10.1177/1073858403009003012. 15065815 10.1177/1073858403009003012

[29] Kimberley TJ, Prudente CN, Engineer ND, Dickie DA, Bisson TA, de Van Winckel A. Vagus nerve stimulation paired with mobility training in chronic ischemic stroke: a case report. Phys Ther. 2023;103(12):pzad097. 10.1093/ptj/pzad097. 37669130 10.1093/ptj/pzad097 PMC10748760

[30] Yan L, Qian Y, Li H. Transcutaneous vagus nerve stimulation combined with rehabilitation training in the intervention of upper limb movement disorders after stroke: a systematic review. Neuropsychiatr Dis Treat. 2022;18:2095–106. 10.2147/NDT.S376399. 36147448 10.2147/NDT.S376399 PMC9488604

[31] Khan F, Chevidikunnan MF. Prevalence of balance impairment and factors associated with balance among patients with stroke: a cross-sectional retrospective case-control study. Healthcare. 2021. 10.3390/healthcare9030320. 33805643 10.3390/healthcare9030320 PMC7998930

[32] Rossi S, Hallett M, Rossini PM, Pascual-Leone A. Screening questionnaire before TMS: an update. Clin Neurophysiol. 2010;122(8):1686. 10.1016/j.clinph.2010.12.037. 10.1016/j.clinph.2010.12.037 21227747

[33] Billingham SAM, Whitehead AL, Julious SA. An audit of sample sizes for pilot and feasibility trials being undertaken in the United Kingdom registered in the United Kingdom Clinical Research Network database. BMC Med Res Methodol. 2013;13:104. 10.1186/1471-2288-13-104. 23961782 10.1186/1471-2288-13-104 PMC3765378

[34] Eldridge SM, Chan CL, Campbell MJ, Bond CM, Hopewell S, Thabane L, et al. CONSORT 2010 statement: extension to randomised pilot and feasibility trials. BMJ. 2016;355:i5239. 10.1136/bmj.i5239. 27777223 10.1136/bmj.i5239 PMC5076380

[35] Capilupi MJ, Kerath SM, Becker LB. Vagus nerve stimulation and the cardiovascular system. Cold Spring Harb Perspect Med. 2020;10(2):a034173. 10.1101/cshperspect.a034173. 31109966 10.1101/cshperspect.a034173 PMC6996447

[36] Kim AY, Marduy A, de Melo PS, Gianlorenco AC, Kim CK, Choi H, et al. Safety of transcutaneous auricular vagus nerve stimulation (taVNS): a systematic review and meta-analysis. Sci Rep. 2022;12(1):22055. 10.1038/s41598-022-25864-1. 36543841 10.1038/s41598-022-25864-1 PMC9772204

[37] Suk WC, Kim SJ, Chang DS, Lee HY. Characteristics of stimulus intensity in transcutaneous vagus nerve stimulation for chronic tinnitus. J Int Adv Otol. 2018;14(2):267–72. 10.5152/iao.2018.3977. 30256201 10.5152/iao.2018.3977 PMC6354472

[38] Holden MK, Gill KM, Magliozzi MR, Nathan J, Piehl-Baker L. Clinical gait assessment in the neurologically impaired: reliability and meaningfulness. Phys Ther. 1984;64(1):35–40. 10.1093/ptj/64.1.35. 6691052 10.1093/ptj/64.1.35

[39] Likert R. Technique for the measurement of attitudes. New York: Archives of Psychology; 1932.

[40] Wade DT, Wood VA, Hewer RL. Recovery after stroke: the first 3 months. J Neurol Neurosurg Psychiatry. 1985;48(1):7–13. 10.1136/jnnp.48.1.7. 3973623 10.1136/jnnp.48.1.7 PMC1028175

[41] Tyson S, Connell L. The psychometric properties and clinical utility of measures of walking and mobility in neurological conditions: a systematic review. Clin Rehabil. 2009;23(11):1018–33. 10.1177/0269215509339004. 19786420 10.1177/0269215509339004

[42] Flansbjer U-B, Holmbäck AM, Downham D, Patten C, Lexell J. Reliability of gait performance tests in men and women with hemiparesis after stroke. J Rehabil Med. 2005;37(2):75–82. 10.1080/16501970410017215. 15788341 10.1080/16501970410017215

[43] da Cristina Silva L, de Danielli Coelho Moraes Faria C, da Cruz Peniche P, de Ayessa Ferreira Brito S, Tavares Aguiar L. Validity of the two-minute walk test to assess exercise capacity and estimate cardiorespiratory fitness in individuals after stroke: a cross-sectional study. Top Stroke Rehabil. 2024;31(2):125–34. 10.1080/10749357.2023.2217639. 37243679 10.1080/10749357.2023.2217639

[44] Kosak M, Smith T. Comparison of the 2-, 6-, and 12-minute walk tests in patients with stroke. J Rehabil Res Dev. 2005;42(1):103–7. 10.1682/jrrd.2003.11.0171. 15742254 10.1682/jrrd.2003.11.0171

[45] Huang M, Pang MY. Psychometric properties of Brief-Balance Evaluation Systems Test (Brief-BESTest) in evaluating balance performance in individuals with chronic stroke. Brain Behav. 2017;7(3):e00649. 10.1002/brb3.649. 28293482 10.1002/brb3.649 PMC5346529

[46] Banks JL, Marotta CA. Outcomes validity and reliability of the Modified Rankin Scale: implications for stroke clinical trials. Stroke. 2007;38(3):1091–6. 10.1161/01.STR.0000258355.23810.c6. 17272767 10.1161/01.STR.0000258355.23810.c6

[47] Jones F, Partridge C, Reid F. The Stroke Self-Efficacy Questionnaire: measuring individual confidence in functional performance after stroke. J Clin Nurs. 2008;17(7B):244–52. 10.1111/j.1365-2702.2008.02333.x. 18578800 10.1111/j.1365-2702.2008.02333.x

[48] Wu SY, Li YC, Chen YW, Chen CL, Pan HC, Lin KC, et al. Construct validity, responsiveness, minimal detectable change, and minimal clinically important difference of the stroke self-efficacy questionnaire in individuals receiving stroke rehabilitation. Disabil Rehabil. 2024. 10.1080/09638288.2024.2324122. 38433459 10.1080/09638288.2024.2324122

[49] Hiengkaew VP, Jitaree KM, Chaiyawat PP. Minimal detectable changes of the Berg Balance Scale, Fugl-Meyer Assessment Scale, Timed Up & go test, gait speeds, and 2-minute walk test in individuals with chronic stroke with different degrees of ankle plantarflexor tone. Arch Phys Med Rehabil. 2012;93(7):1201–8. 10.1016/j.apmr.2012.01.014. 22502805 10.1016/j.apmr.2012.01.014

[50] Lewek MD, Sykes R. Minimal detectable change for gait speed depends on baseline speed in individuals with chronic stroke. J Neurol Phys Ther. 2019;43(2):122–7. 10.1097/NPT.0000000000000257. 30702510 10.1097/NPT.0000000000000257 PMC6361716

[51] Nasreddine ZS, Phillips NA, Bédirian V, Charbonneau S, Whitehead V, Collin I, et al. The Montreal Cognitive Assessment, MoCA: a brief screening tool for mild cognitive impairment. J Am Geriatr Soc. 2005;53(4):695–9. 10.1111/j.1532-5415.2005.53221.x. 15817019 10.1111/j.1532-5415.2005.53221.x

[52] Bang H, Flaherty SP, Kolahi J, Park J. Blinding assessment in clinical trials: a review of statistical methods and a proposal of blinding assessment protocol. Clin Res Regul Aff. 2010;27(2):42–51. 10.3109/10601331003777444.

[53] IBM Corp. IBM SPSS Statistics for Windows, Version 28.0. Armonk (NY): IBM Corp; 2021.

[54] Sullivan GM, Feinn R. Using effect size—or why the P value is not enough. J Grad Med Educ. 2012;4(3):279–82. 10.4300/jgme-d-12-00156.1. 23997866 10.4300/JGME-D-12-00156.1 PMC3444174

[55] Lakens D. Calculating and reporting effect sizes to facilitate cumulative science: a practical primer for t-tests and ANOVAs. Front Psychol. 2013;4:863. 10.3389/fpsyg.2013.00863. 24324449 10.3389/fpsyg.2013.00863 PMC3840331

[56] Chang JL, Coggins AN, Saul M, Paget-Blanc A, Straka M, Wright J, et al. Transcutaneous auricular vagus nerve stimulation (tAVNS) delivered during upper limb interactive robotic training demonstrates novel antagonist control for reaching movements following stroke. Front Neurosci. 2021;15:767302. 10.3389/fnins.2021.767302. 34899170 10.3389/fnins.2021.767302 PMC8655845

[57] Badran BW, Peng X, Baker-Vogel B, Hutchison S, Finetto P, Rishe K, et al. Motor activated auricular vagus nerve stimulation as a potential neuromodulation approach for post-stroke motor rehabilitation: a pilot study. Neurorehabil Neural Repair. 2023;37(6):374–83. 10.1177/15459683231173357. 37209010 10.1177/15459683231173357 PMC10363288

[58] Capone F, Miccinilli S, Pellegrino G, Zollo L, Simonetti D, Bressi F, et al. Transcutaneous vagus nerve stimulation combined with robotic rehabilitation improves upper limb function after stroke. Neural Plast. 2017;2017:7876507. 10.1155/2017/7876507. 29375915 10.1155/2017/7876507 PMC5742496

[59] Redgrave JN, Moore L, Oyekunle T, Ebrahim M, Falidas K, Snowdon N, et al. Transcutaneous auricular vagus nerve stimulation with concurrent upper limb repetitive task practice for poststroke motor recovery: a pilot study. J Stroke Cerebrovasc Dis. 2018;27(7):1998–2005. 10.1016/j.jstrokecerebrovasdis.2018.02.056. 29580658 10.1016/j.jstrokecerebrovasdis.2018.02.056

[60] Meints SM, Garcia RG, Schuman-Olivier Z, Datko M, Desbordes G, Cornelius M, et al. The effects of combined respiratory-gated auricular vagal afferent nerve stimulation and mindfulness meditation for chronic low back pain: a pilot study. Pain Med. 2022. 10.1093/pm/pnac025. 35148407 10.1093/pm/pnac025 PMC9434172

[61] Napadow V, Edwards RR, Cahalan CM, Mensing G, Greenbaum S, Valovska A, et al. Evoked pain analgesia in chronic pelvic pain patients using respiratory-gated auricular vagal afferent nerve stimulation. Pain Med. 2012;13(6):777–89. 22568773 10.1111/j.1526-4637.2012.01385.x PMC3376238

[62] Paccione CE, Stubhaug A, Diep LM, Rosseland LA, Jacobsen HB. Meditative-based diaphragmatic breathing vs. vagus nerve stimulation in the treatment of fibromyalgia: body vs. machine. Front Neurol. 2022;13:1030927. 36438970 10.3389/fneur.2022.1030927 PMC9687386

[63] Shi X, Zhao L, Luo H, Deng H, Wang X, Ren G et al. Transcutaneous auricular vagal nerve stimulation is effective for the treatment of functional dyspepsia: a multicenter, randomized controlled study. Am J Gastroenterol. 2023. 10.14309/ajg.0000000000002548 37787432

[64] Stavrakis S, Elkholey K, Morris L, Niewiadomska M, Asad ZUA, Humphrey MB. Neuromodulation of inflammation to treat heart failure with preserved ejection fraction: a pilot randomized clinical trial. J Am Heart Assoc. 2022;11(3):e023582. 35023349 10.1161/JAHA.121.023582 PMC9238491

[65] Wu Z, Zhang X, Cai T, Li Y, Guo X, Zhao X, et al. Transcutaneous auricular vagus nerve stimulation reduces cytokine production in sepsis: an open double-blind, sham-controlled, pilot study. Brain Stimul. 2023;16(2):507–14. 36801260 10.1016/j.brs.2023.02.008

[66] Storm FA, Buckley CJ, Mazzà C. Gait event detection in laboratory and real life settings: accuracy of ankle and waist sensor based methods. Gait Posture. 2016;50:42–6. 10.1016/j.gaitpost.2016.08.012. 27567451 10.1016/j.gaitpost.2016.08.012

[67] Badran BW, Huffman SM, Dancy M, Austelle CW, Bikson M, Kautz SA, et al. A pilot randomized controlled trial of supervised, at-home, self-administered transcutaneous auricular vagus nerve stimulation (taVNS) to manage long COVID symptoms. Res Sq. 2022. 10.21203/rs.3.rs-1716096/v1. 36002874 10.1186/s42234-022-00094-y PMC9402278

