# Supplementary material for: Transcutaneous auricular vagus nerve stimulation paired with task-specific training for improving walking and balance in chronic stroke: a double-blind, randomised controlled feasibility trial
Source: J Neuroeng Rehabil. 2026 Jun 25;23:231. doi: 10.1186/s12984-026-01952-5 (PMC13455390; doi:10.1186/s12984-026-01952-5)
Supplement: Supplementary file 1 [file 12984_2026_1952_MOESM1_ESM.docx]

**Adverse events questionnaire**

Adapted from Gerges ANH, Williams EER, Hillier S, Uy J, Hamilton T, Chamberlain S, Hordacre B. Clinical application of transcutaneous auricular vagus nerve stimulation: a scoping review. Disabil Rehabil. 2024 Feb 16:1-31. doi: 10.1080/09638288.2024.2313123. Epub ahead of print. PMID: 38362860.

**Blinding questionnaire**

**Transcript to assist with blinding participants**

" In this study, we will use different stimulation parameters for different groups of participants and depending on the parameters used in your intervention group, you may or may not feel the stimulus”.

Table: Feasibility Criteria

|  | Proceed | Proceed with Protocol Amendments | Significant Amendments Required |
| --- | --- | --- | --- |
| Criteria | 50% of eligible participants consent to participate | 40% of eligible participants consent to participate | 25% of eligible participants consent to participate |
|  | 60% of participants complete the intervention protocol | 40% of participants complete the intervention protocol | 25% of participants complete the intervention protocol |
|  | 50% found intervention useful (Likert ≥ 4/5) | 25% found intervention useful (Likert ≥ 4/5) | < 25% found intervention useful (Likert ≥ 4/5) |
|  | 50% found intervention helpful (Likert ≥ 4/5) | 25% found intervention helpful (Likert ≥ 4/5) | < 25% found intervention helpful (Likert ≥ 4/5) |
|  | 50% found intervention delivery acceptable (Likert ≥ 4/5) | 25% found intervention delivery acceptable (Likert ≥ 4/5) | < 25% found intervention delivery acceptable (Likert ≥ 4/5) |
|  | Blinding: ≥ 90% of participants were blinded | Blinding: ≥ 50% of participants were blinded | Blinding: < 50% of participants were blinded |
|  | Minimal AEs occur in < 20 % of participants. | Minimal AEs occur in ≥ 20% and < 50 %, and no severe AEs. | Minimal AEs occur in ≥ 50, and/or severe AEs occur in any participants. |

**Interview Guide**

Please note that this interview is being recorded and will be transcribed for analysis. Do you consent to this?

**Qu 1:** How did you find your experience with using the non invasive auricular vagus nerve stimulation?

**Qu 2**: What things did you like about using non invasive auricular vagus nerve stimulation?

What things didn’t you like about using non invasive auricular vagus nerve stimulation?

**Qu 3:** For the therapy sessions you were not able to complete, what would you say was the major factor preventing you from doing so?

**Qu 4:** Do you think your balance and mobility has changed since your stroke? In what way has it changed and do you think it was because of the non invasive auricular vagus nerve stimulation?

**Qu 5**: Has your participation in activities changed since your stroke? What is your opinion about the role of non invasive auricular vagus nerve stimulation in these changes?

**Qu 6**: If you were given the opportunity would you continue to use taVNS after this treatment finished?

**Qu 7:** How would you rate the comfort/ability to tolerate the device/stimulation?

**Qu 8:** Do you think using equipment such as non invasive auricular vagus nerve stimulator in the future in clinics or at home would be worthwhile?

**Qu 9:** Are there any other comments you would like to make about your participation and experience in the trial?

**Table:** Summary of reported AEs related to taVNS

| **AE** | **Participants**  **N** | **AEs**  **N** | **Severity** | **Duration of symptoms** | **Participants**  **N /%** |
| --- | --- | --- | --- | --- | --- |
| **Irritation** | 1 | 3 | Mild | Resolved immediately when stimulation ended. | 4 (16%) |
| **Itchiness** | 1 | 2 |  |  |  |
| **Pain** | 2 | 2 |  |  |  |
| **Headaches** | 1 | 3 |  | Resolved within 1-2 hours of ending stimulation. |  |
| **Fatigue** | 1 | 1 |  | Resolved once stimulation ended |  |
| **Dizziness/**  **lightheadedness** | 1 | 1 |  | Intermittent during the stimulation lasting for minutes and resolved completely when stimulation ended. |  |

**Legend:** AE; Adverse Event, N = number. * Note: two participants reported more than one symptom.

**Table: Blinding data**

| **Intervention** | **Primary guesses  N** | | | | **Forced guesses N** | |
| --- | --- | --- | --- | --- | --- | --- |
|  | **Real** | **Sham** | **"Don’t know"** | **Total** | **Real** | **Sham** |
| **Active taVNS** | 8 | 1 | 5 | 14 | 2 | 3 |
| **Sham taVNS** | 2 | 3 | 6 | 11 | 2 | 4 |

**Legend:** taVNS**:** transcutanous auricular vagus nerve stimulation, N: number

# **Table:** Comparison between clinical outcome measures for male and female participants in the active taVNS group, post-intervention.

| **Measure** | **t** | **df** | **Sig. Two-sided p** | **Mean difference** | **Std. error difference** | **95% Confidence interval of the difference** | |
| --- | --- | --- | --- | --- | --- | --- | --- |
|  |  |  |  |  |  | **Lower** | **Upper** |
| 10MWT (fast-paced m/sec) | 0.23 | 12 | 0.82^a^ | 0.05 | 0.22 | -0.43 | 0.53 |
| 2MWT (m) | -0.01 | 12 | 0.99^a^ | -0.32 | 22.01 | -48.27 | 47.64 |
| Self-paced gait speed (m/sec) | 1.02 | 10 | 0.33^a^ | 0.112 | 0.11 | -0.14 | 0.36 |
| Cadence (steps/min) | 0.34 | 10 | 0.74^a^ | 1.63 | 4.83 | -9.14 | 12.41 |
| Brief BESTest | -0.73 | 12 | 0.48^a^ | -2.33 | 3.21 | -9.34 | 4.68 |
| SSEQ | -0.8 | 12 | 0.44^a^ | -15.73 | 19.75 | -58.75 | 27.3 |
| mRS | NA | NA | 1.00^b^ | NA | NA | NA | NA |

**Legend:** taVNS: transcutaneous auricular vagus nerve stimulation were used to compare the mean difference between baseline and post-intervention between males and females. SSEQ: Stroke self-efficacy questionnaire, BESTest: Balance evaluation system test, 10MWT: 10-meter walk test, 2MWT: 2-minute walk test, mRS: Modified Rankin scale, NA: not applicable. ^a^ Independent t-test, and b Fisher’s Exact test.

# **Table:** Comparison of clinical outcome measures between participants with left-hemisphere and right-hemisphere brain lesions in the active taVNS group, post-intervention.

| **Measure** | **t** | **df** | **Two-Sided p** | **Mean Difference** | **Std. Error Difference** | **95% Confidence Interval of the Difference** | |
| --- | --- | --- | --- | --- | --- | --- | --- |
|  |  |  |  |  |  | **Lower** | **Upper** |
| **10MWT (fast-paced m/sec)** | -0.46 | 12 | 0.66^a^ | -0.05 | 0.10 | -0.27 | 0.18 |
| **2MWT (m)** | -0.05 | 12 | 0.96^a^ | -0.65 | 12.92 | -28.80 | 27.51 |
| **Self-paced gait speed (m/sec)** | 1.16 | 12 | 0.27^a^ | 0.13 | 0.11 | -0.12 | 0.38 |
| **Cadence (steps/min)** | 0.69 | 11 | 0.51^a^ | 3.14 | 4.57 | -6.91 | 13.19 |
| **Brief BESTest** | -1.23 | 12 | 0.24^a^ | -2.54 | 2.06 | -7.03 | 1.95 |
| **SSEQ** | -1.41 | 12 | 0.18^a^ | -7.20 | 5.12 | -18.35 | 3.94 |
| **mRS** | NA | NA | 1.00^b^ | NA | NA | NA | NA |

Legend: taVNS: transcutaneous auricular vagus nerve stimulation were used to compare the mean difference between baseline and post-intervention between males and females. SSEQ: Stroke self-efficacy questionnaire, BESTest: Balance evaluation system test, 10MWT: 10-meter walk test, 2MWT: 2-minute walk test, mRS: Modified Rankin scale, NA: not applicable. ^a^ Independent t-test, and b Fisher’s Exact test.
